# Gestational or acute restraint in adulthood reduces levels of 5α-reduced testosterone metabolites in the hippocampus and produces behavioral inhibition of adult male rats

**DOI:** 10.3389/fncel.2012.00040

**Published:** 2012-12-18

**Authors:** Alicia A. Walf, Cheryl A. Frye

**Affiliations:** ^1^Life Sciences Research, The University at Albany-SUNYAlbany, NY, USA; ^2^Departments of Psychology, The University at Albany-SUNYAlbany, NY, USA; ^3^Biological Sciences, The University at Albany-SUNYAlbany, NY, USA; ^4^The Centers for Neuroscience, The University at Albany-SUNYAlbany, NY, USA

**Keywords:** testosterone, androgens, anxiety, affect, inhibitory avoidance, prenatal stress

## Abstract

Stressors, during early life or adulthood, can alter steroid-sensitive behaviors, such as exploration, anxiety, and/or cognitive processes. We investigated if exposure to acute stressors in adulthood may alter behavioral and neuroendocrine responses of male rats that were exposed to gestational stress or not. We hypothesized that rats exposed to gestational and acute stress may show behavioral inhibition, increased corticosterone, and altered androgen levels in the hippocampus. Subjects were adult, male offspring of rat dams that were restrained daily on gestational days 14–20, or did not experience this manipulation. Immediately before testing, rats were restraint stressed for 20 min or not. During week 1, rats were tested in a battery of tasks, including the open field, elevated plus maze, social interaction, tailflick, pawlick, and defensive burying tasks. During week 2, rats were trained and tested 24 h later in the inhibitory avoidance task. Plasma corticosterone and androgen levels, and hippocampal androgen levels, were measured in all subjects. Gestational and acute restraint stress increased plasma levels of corticosterone, and reduced levels of testosterone's 5α-reduced metabolites, dihydrotestosterone (DHT) and 3α-androstanediol (3α-diol), but not the aromatized metabolite, estradiol (E_2_), in plasma or the hippocampus. Gestational and acute restraint stress reduced central entries made in the open field, and latencies to enter the shock-associated side of the inhibitory avoidance chamber during testing. Gestational stress reduced time spent interacting with a conspecific. These data suggest that gestational and acute restraint stress can have actions to produce behavioral inhibition coincident with increased corticosterone and decreased 5α-reduced androgens of adult male rats. Thus, gestational stress altered neural circuits involved in the neuroendocrine response to acute stress in early adulthood.

## Introduction

The profound effects of stress on the nervous, immune, metabolic, and cardiovascular systems for health-related outcomes throughout development may depend in part upon the timing of exposure to stressors. On a basic level, acute stress has adaptive short-acting effects on systems that can mobilize individuals from stimuli that challenge homeostasis. Early life, chronic stress has pervasive physiological, neuroendocrine, and behavioral consequences, involving hypothalamic-pituitary-adrenal axis (HPA) dysfunction, that may contribute to pathological conditions [e.g., depression, posttraumatic stress disorder (PTSD), premature aging, hypertension, insulin resistance, etc.; Barker et al., 1993; Bremner et al., 1997; Seckl, 2004; Entringer et al., 2008]. Thus, the nature of stressors' effects may depend upon the timing of exposure.

Animal models of early life stress are useful to elucidate some of the perinatal determinants of adult psychopathology. One valid model of early life stress involves exposing rat dams to restraint stress during late gestation and then assessing the developmental and behavioral outcomes of their offspring. The construct validity of this model of HPA dysfunction is supported by rats exposed to gestational stress having higher baseline and stress-induced corticosterone levels (reviewed in Weinstock, 2007). There is also face validity in this model. There are reports in the clinical literature that gestational stress can produce cognitive impairments and deficits in affective responses, as well as increased risk for diagnoses of psychopathologies, in addition to these alterations in the HPA (reviewed in Weinstock, 2007). Despite the clear validity of this model, the nature of these effects may depend upon sex/gender, developmental stage, and other factors.

Sex differences, or the early organizing role of gonadal steroids, need to be considered in the context of gestational stress. In support, our laboratory, and others, has shown that young adult female rats may experience more deleterious physiological, neuroendocrine, and behavioral effects of gestational stress than do their male littermates (Koehl et al., 1991; Weinstock et al., 1992; McCormick et al., 1995; Alonso et al., 1997; Sternberg, 1999; Szuran et al., 2000; Frye and Wawrzycki, 2003). Furthermore, restraint stress on gestational day 18 decreases the volume of the hippocampus of adult female rats, compared to male littermates as adults, or age-matched, non-prenatally stressed controls (Schmitz et al., 2002). However, not all studies show this pattern of effects. For instance, there are clear effects of gestational stressors among rats to increase anxiety behavior in the elevated plus maze of male, but not female, rats compared to non-stressed controls (Zuena et al., 2008; Brunton and Russell, 2010). An important consideration is the timing of the stressor effects (Andersen and Teicher, 2004). Early gestational stress (days 1–7) produces performance deficits in the Barnes maze of adult male mice compared to that of non-stressed controls; whereas early gestational stress enhances females' performance (Mueller and Bale, 2007). Additionally, male, but not female, mice exposed to stress during gestational days 1–7 have increased depression-like behavior in the tail suspension and forced swim tasks and reduced sucrose preference and greater HPA activity, compared to non-stressed mice (Mueller and Bale, 2008). These effects were not observed in mice that were gestationally stressed during mid to late pregnancy. Another notion to consider is that vulnerability to pervasive effects of early stress may occur at a later stage of development in males than in females. For example, there are sex differences in response to early adversity among rhesus macaques (Cirulli et al., 2009). On postnatal day (PND) 60, but not before as observed in females, male rhesus macaques showed effects of peer-rearing stress (e.g., had increased cortisol and reduced play behavior) compared to mother-reared controls (Cirulli et al., 2009). Overall, these and other data, suggest that there are sex differences and timing effects of stressor exposure for adult stress responding and behavior (Bowman et al., 2004; Bowman, 2005). Androgens have well-known organizing and activating effects on neural and behavioral outcomes. Of interest is the extent to which gestational stress may alter later androgen secretion and androgen-mediated behavioral effects.

Recent studies have suggested that early challenges may alter later secretion and effects of pregnane steroids produced *de novo* in the brain (“neurosteroids”). In support, male and female rats that were exposed to an immune challenge stressor during late gestation had lower levels of a pregnane neurosteroid, 5α-pregnan-3α-ol-20-one (3α,5α-THP), than did control rats when they were assessed at PND 28–30 (Paris et al., 2011a). Moreover, male rats exposed to such a stressor during gestation show a more female-like pattern of anxiety-like behavior in the open field, compared to control male rats, tested at PND 28–30. A similar pattern of effects was observed for restraint stress, variable stressors, or administration of finasteride, a 5α-reductase inhibitor, during late gestation, to produce deficits in object recognition memory and lower pregnane neurosteroids among juvenile male and female rats (Paris and Frye, 2011a,b; Paris et al., 2011a,b). In these studies, androstane neurosteroids were not measured, but finasteride would be expected to similarly reduce levels of androstane neurosteroids. The effects of gestational stress on neurosteroidogenesis persist into adulthood. For example, adult female rats that were exposed to gestational stress have lower hippocampus levels of 3α,5α-THP, as well as increased depressive-like responding in the forced swim test, compared to controls (Frye and Walf, 2004). These studies show clear behavioral deficits coincident with decrements in neuroendocrine responding (i.e., lower pregnane neurosteroids) among gestationally stressed offspring. Although both males and females secrete pregnane neurosteroids, it may be that males are less sensitive to pregnane neurosteroids than to androstane neurosteroids, which are produced at greater levels among males than females.

It is of interest to determine the extent to which some of the effects of gestational stress on male offspring are related to androstane neurosteroids. Some sex differences noted in adult rodents for HPA axis activity may be related to actions of androgens. For example, there is greater activity in brain regions known to inhibit the HPA, such as the hippocampus, among gonadally intact males compared to females or gonadectomized male mice (Goel et al., 2011). Moreover, studies conducted in our laboratory and others have demonstrated that androgens can have activational effects to reduce anxiety- and depression-like behaviors and enhance cognitive performance of adult male rats and mice (Frye and Seliga, 2001; Aikey et al., 2002; Edinger and Frye, 2004; Fernández-Guasti and Martínez-Mota, 2005; Buddenberg et al., 2009). These effects may be due to actions of testosterone (T) and/or its metabolites in the hippocampus. T is aromatized to produce estradiol (E_2_), and metabolized by 5α-reductase and 3α-hydroxysteroid dehydrogenase (3α-HSD) to form dihydrotestosterone (DHT) and 3α-androstanediol (3α-diol), respectively. DHT and 3α-diol are androstane neurosteroids produced locally in the brain, in areas, such as the hippocampus, which has high levels of expression of the requisite enzymes (Tsuruo, 2005). The hippocampus is sensitive to the effects of T metabolites to enhance neurogenesis in adult rats (Spritzer and Galea, 2007; Galea, 2008). There are clear effects of gestational stress on hippocampus structure and/or function of rodents (Schmitz et al., 2002; Kim et al., 2006; Setiawan et al., 2007; Weinstock, 2007). The importance of 3α-diol in the hippocampus for the behavioral effects of androgens has been reported in non-stressed adult male rats (Frye et al., 2010). A question is the role of gestational stress, coinciding with the development of the hippocampus (i.e., late pregnancy), for later androgen responses. We hypothesized that: (1) exposure to gestational stress of male rats during late pregnancy would alter neuroendocrine function (increase corticosterone, decrease androstane neurosteroids in the hippocampus) and behavior (decrease exploration, social interaction, and inhibitory avoidance), (2) there would be similar effect of acute restraint stress in adulthood to increase corticosterone, decrease neurosteroidogesis, and produce behavioral inhibition, and (3) gestational stress may alter later responses to acute restraint stress of adults for these neuroendocrine and behavioral measures.

## Materials and methods

All methods utilized in this study using animal subjects were pre-approved by the Institutional Animal Care and Use Committee at the University at Albany-SUNY.

### Subjects and housing

Gonadally-intact, adult male Long-Evans rats (~55 days of age; 250–300 g) were experimental subjects in this study (*N* = 48). Rats were obtained from our breeder colony (original breeders from Taconic, Germantown, NY) in the Social Sciences Laboratory Animal Care Facility at the University at Albany-SUNY following gestational stress or not (described below). They were group-housed 3–4 per cage with continuous access to Purina Rat Chow and tap water on a 12/12 h reversed light-dark cycle (lights off at 0800 h). Experimental rats were picked up by animal care staff and placed into clean cages once a week.

### Gestational stress

Female breeder rats (*n* = 60) were cycled through two normal, 4–5 day estrous cycles and mated on behavioral estrus. Pregnant rats were then randomly assigned to the control (*n* = 26) or restraint stress (*n* = 34) condition. Rats in the control condition remained undisturbed in their home cages throughout pregnancy, except for weekly cage cleaning by animal care staff. The pregnant rats that were restraint stressed experienced weekly cage cleaning in the same manner as did the control breeders, but were restraint stressed by being placed in a Plexiglas restrainer (7.5 cm diameter × 19.5 cm length) under a 60-watt light for 45 min daily from gestational days 14–20 (Frye and Walf, 2004). Although it was not systematically examined, overt differences in body weight of the dams, or weight or length of the pups, were not apparent. However, this type of chronic stress during gestation can produce profound effects to interfere with reproductive outcomes and reduce fertility, length of gestation, and litter size (Paris and Frye, 2011a,b). Additionally, gestational stress can have long-lasting effects on HPA responding, such that restraint stress from gestational day 17–21 increases corticosterone in dams at the time of birth compared to control dams (Paris and Frye, 2011b). To control for potential litter effects, which may be due to differences in maternal care, one pup from each litter in the control or gestational stress conditions were utilized so that there was not over-representation of any one litter in the experimental groups. Cross-fostering was not utilized as this produced confounds and/or detrimental effects in some studies (Macrì et al., 2010).

### Acute restraint stress

As adults, rats were randomly assigned to be in the control, non-restraint stressed condition (*n* = 24), or they experienced acute restraint stress (*n* = 24). Restraint stress consisted of placing rats in Plexiglas restrainers (7.5 cm diameter × 19.5 cm length) for 20 min, under a 60-watt light (Walf and Frye, 2005). Temperatures of the restraint tube, when placed 30.5 cm under such a lamp, rise from 20 to 21°C within 1 min and remain at this temperature 20 and 45 min later. As such, this is not considered a heat stressor when utilized for 45 min as a gestational stressor, or when utilized for 20 min as an acute stressor in adults. We have verified that this acute restraint stress protocol increases corticosterone levels following restraint stress and 20 min of behavioral testing (open field, elevated plus maze, forced swim test) compared to behavioral testing in these tasks alone among female rats (Walf and Frye, 2005). Thus, there were four experimental conditions (*n* = 12/condition): (1) Non-gestationally stressed, non-acute stressed control, (2) Non-gestationally stressed, acute restraint stressed, (3) Gestationally stressed, non-acute stressed, (4) Gestationally stressed, acute restraint stressed.

### Behavioral testing

Traditional measures of stress/anxiety of rodents were utilized as behavioral indices of hippocampal function (open field, elevated plus maze, defensive freezing) and hippocampal/amygdala function (inhibitory avoidance task). Because footshock was utilized as stimuli, pain thresholds (tailflick and pawlick latencies) were assessed. Handling can alter behavioral responses, so rats received the same amount of handling before testing. Each rat was picked up once each week by the animal care staff for cage changing, and then consistently picked up by the experimenter immediately before behavioral testing. Rats had behavioral assessments in a battery of tasks (open field, elevated plus maze, social interaction, tailflick, pawlick, and defensive freezing) during the first week of testing. The next week, rats were habituated and trained in the inhibitory avoidance task and then tested the following day. All behavioral tasks were run by observers blind to the hypothesized outcome of the study and/or gestational stress condition of rats. Testing chambers were thoroughly cleaned with dilute Quatricide and dried with paper towels between each test. The bars on the grid floor of the inhibitory avoidance chamber were also cleaned with 70% isopropyl alcohol.

#### Open field

Rats were placed in the southeast corner of the open field. Entries into central and peripheral squares of the open field (76 × 57 × 35 cm) were recorded during the 5-min task. Entries were defined by placement of all four paws in the square. The total and central square entries made in the open field are utilized as indices of general motor/exploratory and reduced anxiety-like behavior, respectively (Walf and Frye, 2005).

#### Elevated plus maze

In the elevated plus maze, rats were placed in the junction of the four arms (two alleyways with walls, and two alleyways without walls) of the elevated plus maze (Walf and Frye, 2005). The time spent by rats on the open and closed arms was recorded during this 5-min task. Open arm time is considered an index of reduced anxiety-like behavior.

#### Social interaction

A stimulus male from the breeder colony that was gonadally intact was placed in the open field for this task. This male rat had been habituated to this task and similar tasks so that the behavior of the experimental animal did not depend upon that of the stimulus male conspecific. The time spent by the experimental rat engaging in social interaction with the conspecific (with the experimental male making the contact) was recorded during this 5 min task (Frye and Seliga, 2001). Social interaction was defined by grooming, sniffing, touching, and following with contact when it was initiated by the experimental rat. The time spent in social interaction is utilized as a measure of social behavior.

#### Pawlick task

The latency for rats to lick their front or back paws following placement on a heated surface (Fisherbrand test tube warmer; 50°C) was recorded (Frye and Seliga, 2001). The maximum latency in this task was 180 s. This latency is utilized as an index of anti-nociceptive behavior in this task.

#### Tailflick task

The latency for rats to reflexively move their tails from a heat source (San Diego Instruments; 50°C) was recorded for three consecutive trials and averaged (Frye and Seliga, 2001). The maximum latency for each trial was 10 s. The average tailflick latency is utilized as an index of anti-nociceptive behavior in this task.

#### Defensive burying task

Rats were tested in the defensive burying task as per published methods (Frye and Seliga, 2001). Rats were placed in the southeast corner of a testing chamber (clear Plexiglas, 26.0 × 21.2 × 24.7 cm, with woodchip bedding). In the northwest corner of the chamber, there was a cylindrical pedestal (2.5 cm diameter, 10.0 cm height) wrapped by wires connected to a shock source (Lafayette Model A615B, Lafayette, IN) set to deliver 0.66 mA of unscrambled shock. When rats touched the pedestal, a brief footshock was delivered, which was terminated immediately following the rat's withdrawal of its paw from the pedestal. The duration spent burying the pedestal with the woodchip bedding in response to the footshock received by the rat was recorded for 15 min following shock. The time spent burying was utilized as an index of anxiety-like responding.

#### Inhibitory avoidance task

The inhibitory avoidance task was conducted in accordance with previously published methods (Edinger and Frye, 2004). The chamber consisted of two compartments (a white illuminated compartment and a black dark compartment) divided by a guillotine door. All rats were habituated for 2 min on the white side of the box. During training rats were placed into the white side of the box for 1 min before the guillotine door was lifted by the experimenter. The latency for rats to crossover to the dark side of the chamber (max. latency 20 min) was recorded and the door was closed. Rats were then administered a mild footshock (0.2 mA, 5 s duration) through a grid floor, and left in the dark side of the chamber for 1 min. The next day, rats were placed in the white chamber for 1 min, the door was lifted, and the latency to move to the dark side was recorded (max latency 5 mins). Longer crossover latencies indicate better inhibitory avoidance performance.

### Measurement of corticosterone and androgen levels

#### Tissue collection, storage, and preparation for radioimmunoassay

After testing in the inhibitory avoidance task, rats were rapidly decapitated and trunk blood and whole brains were collected. Blood was spun in a refrigerated centrifuge at 3000 g at 4°C. Whole brains were rapidly frozen on dry ice immediately after dissection from the skull. Tissues were placed in long-term storage in a −80°C freezer. Brains were thawed on weigh boats placed on ice and the entire hippocampus was dissected out. Hippocampus samples were homogenized with a glass/Teflon homogenizer in distilled water and trace amounts of [^3^H] steroid.

#### Steroid extraction for radioimmunoassay

Steroids were extracted as follows to measure corticosterone in plasma and androgens (T, E_2_, DHT, and 3α-diol) in plasma and hippocampus (Edinger and Frye, 2004; Frye et al., 2010). Corticosterone was extracted from 10 μl of plasma by heating plasma samples at 60°C for 30 min. Plasma samples for extraction of E_2_, T, DHT, and 3α-diol were incubated at room temperature with distilled water and 800 cpms of [^3^H] steroid. Plasma samples were then snap frozen twice by placing an acetone bath with dry ice, and then test tubes were placed in a savant to evaporate ether. Dried down samples were reconstituted by adding the same volume of 0.1 M phosphate assay buffer (pH 7.4) as the original plasma volume immediately before set-up of radioimmunoassays. Androgens were extracted from the hippocampus homogenate with diethyl ether, which was subsequently evaporated. Samples were reconstituted in 0.1 M phosphate assay buffer (pH 7.4).

#### Radioimmunoassay of corticosterone and androgens

Typical radioimmunoassay methods for plasma corticosterone and plasma and brain androgens were employed (Edinger and Frye, 2004; Frye et al., 2010). The range of the standard curves, prepared in duplicate, was 0–4 ng for corticosterone, 12.5–1000 for E_2_ 50–2000 pg for T and DHT and 0–2000 pg for 3α-diol. Samples were added to assay buffer followed by addition of the appropriate antibody and [^3^H] steroid (PerkinElmer). For corticosterone measurement, samples were incubated at room temperature for 60 min with [^3^H] corticosterone (NET 182: specific activity = 48.2 ci/mmol; New England Nuclear) and corticosterone antibody (B#3-163; Esoterix Endocrinology, Calabasas Hills, CA), which binds 40–60% of corticosterone at a 1:20,000 concentration. T, DHT, and 3α-diol assays were incubated overnight at 4°C. E_2_ was incubated for 60 mins at room temperature. The E_2_ antibody (#244; Dr. Niswender, Colorado State University, Fort Collins, CO) binds approximately 90% of [^3^H] E_2_ (NET-317, 51.3 Ci/mmol) in a 1:40,000 dilution. The T antibody (T3–125; Esoterix Endocrinology) only has modest cross reactivity with DHT, and binds between 60 and 65% of [^3^H] T (NET-387: specific activity = 51.0 ci/mmol) in a 1:20,000 dilution. The DHT antibody (DT3-351; Esoterix Endocrinology; 1:20,000 dilution) is moderately specific to DHT, but there is some cross-reactivity with T and binds 60–65% of [^3^H] DHT (NET-302; specific activity = 43.5 Ci/mmol). The 3α-diol antibody (X-144; Dr. P.N. Rao, Southwest Foundation for Biomedical Research, San Antonio, TX) is highly specific to 3α-diol and binds about 96% of [^3^H] 3α-diol (NET-806: specific activity = 41.00 ci/mmol) when used in a 1:20,000 dilution. Dextran-coated charcoal in assay buffer was rapidly added to assay tubes and samples were incubated with charcoal for 20 min. Samples were then spun in a refrigerated centrifuge at 3000 g at 4°C for 20 min to separate bound and free steroid. Supernatant was decanted into a glass scintillation vials with 5 ml Scintiverse BD (Fisher Scientific). Total assay volumes were 950 μl for corticosterone and 1200 μl for androgens. The concentration of the samples was determined by using the logit-log method of Rodbard and Hutt (1974), interpolation of the standards, and correction for recovery with Assay Zap. Intra- and inter-assay coefficients of variance for these assays are: corticosterone: 5% and 8%; T: 5% and 5%; E_2_: 8% and 10%; DHT: 2% and 10%; 3α-diol: 9% and 10%.

### Data analyses

A MANOVA was utilized to determine the extent to which there was a pattern of the independent variables of stressor exposure for behavior across the several tasks that were utilized. These results suggested that there was a difference between measures utilized, with the most robust effects noted in the tasks that rats were exposed to immediately after restraint stress (open field, inhibitory avoidance) or a highly androgen sensitive task (social interaction). Two-way analyses of variance (ANOVAs) with Fisher's *post-hoc* tests were used to evaluate effects of gestational and acute restraint stress on behavioral indices and steroid levels. Given evidence for the nature and timing of the task to influence outcomes, we will focus the discussion of the results on the open field, inhibitory avoidance, and social interaction tasks. As a proxy of metabolism enzyme activity, ratios of the metabolites, DHT and 3α-diol, to the parent hormone, T, in the hippocampus were calculated and compared. The α level for statistical significance was *p* < 0.05, and a trend was considered when *p* < 0.10.

## Results

### Effects of gestational stress

**Figure 1:**
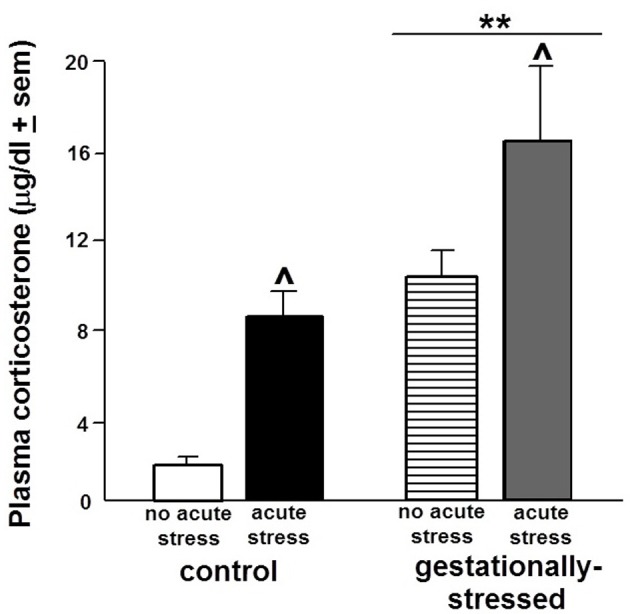
**Plasma Corticosterone Levels.** Figure depicts the plasma levels of corticosterone (mean ± s.e.m.) of adult male rats that were gestationally stressed or not, and then restraint stressed, or not, immediately before testing in the open field. ^**^Above line indicates a significant difference of gestational stress compared to non-gestationally stressed (control) rats (*p* < 0.05 for main effect and Fisher's PLSD *post-hoc* tests). ^∧^ Indicates a significant difference of acute restraint stress compared to no acute stress group (*p* < 0.05 for main effect and Fisher's PLSD *post-hoc* tests). There was no significant interaction between stress variables for plasma corticosterone levels.

Gestational stress had pervasive effects to alter HPA responding as demonstrated by a main effect of gestational stress on plasma corticosterone levels [*F*_(1, 44)_ = 16.87, *p* < 0.01]. *Post hoc* analyses demonstrated that rats exposed to gestational stress had significantly higher plasma corticosterone levels than did non-stressed, control rats (Figure 1). Corticosterone levels in the non-stressed control group were akin perhaps to those reportedly in similarly non-stressed adult male rats (e.g., 2.9 μg/dl ± 1.3 s.e.m.; Frye et al., 2010).

**Figure 2:**
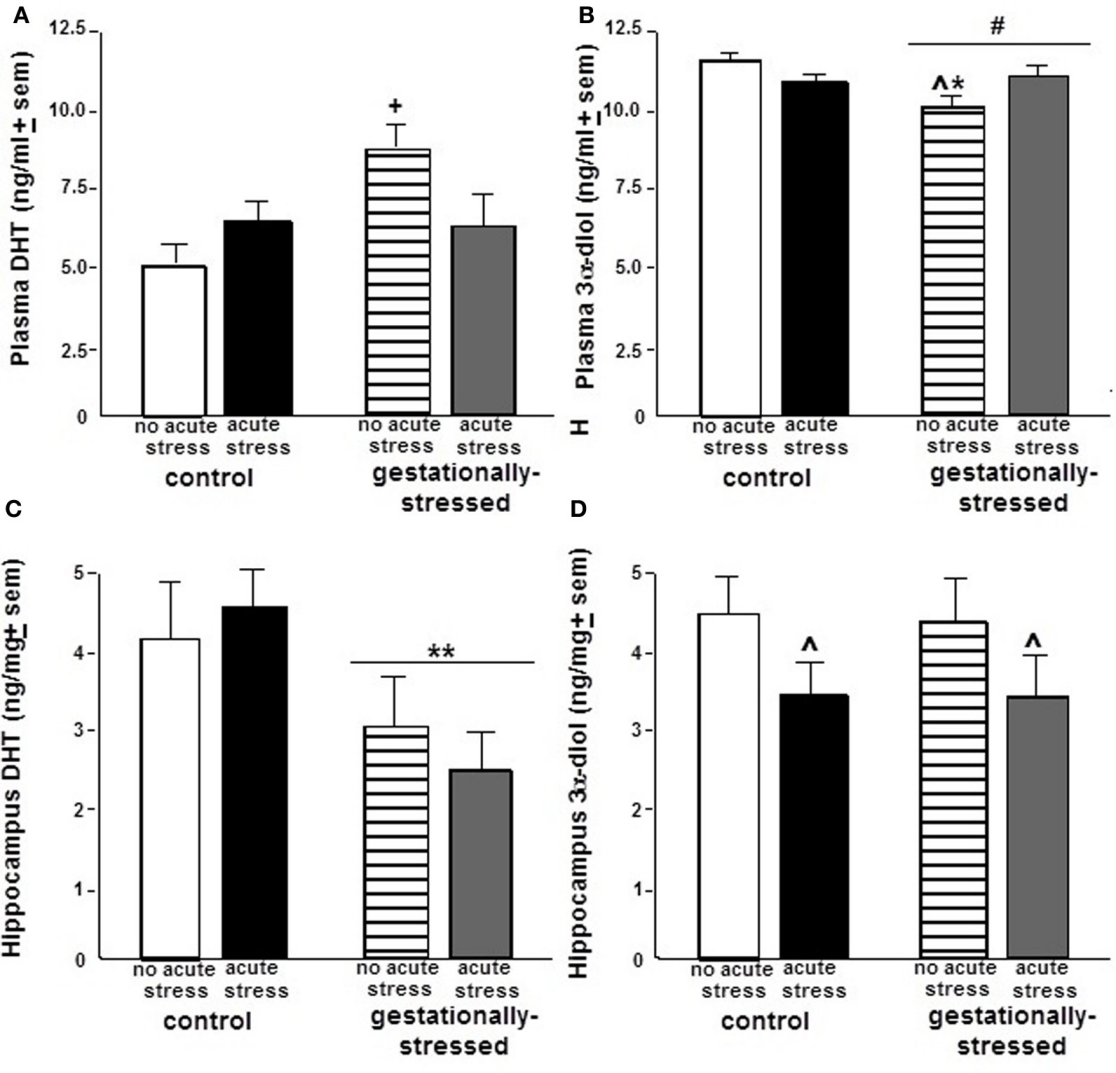
**Plasma and Hippocampal Dihydrotestosterone and 3α-Androstanediol Levels.** Figure depicts the plasma levels (mean ± s.e.m.) of dihydrotestosterone (DHT; panel **A**) and 3α-androstanediol (3α-diol; panel **B**) and hippocampal levels (mean ± s.e.m.) of DHT (panel **C**) and 3α-diol (panel **D**) of adult male rats that were gestationally stressed or not, and then restraint stressed, or not, immediately before testing in the open field. ^**^ Above line indicates a significant effect of gestational stress vs. no gestational stress (*p* < 0.05 for main effect and Fisher's PLSD *post-hoc* tests). ^∧^ Indicates a significant effect of restraint stress vs. no acute stress (*p* < 0.05 for main effect and Fisher's PLSD *post-hoc* tests). ^*∧^ Indicates an interaction between gestational and restraint stress (*p* < 0.05 for main effect and Fisher's PLSD *post-hoc* tests). ^+^ Indicates a tendency for an interaction between gestational and restraint stress (*p* < 0.10 for main effect and *p* < 0.05 for Fisher's PLSD *post-hoc* tests). ^#^ Indicates a tendency for difference of gestational stress compared to non-stress control condition (*p* < 0.10 for main effect and *p* < 0.05 for Fisher's PLSD *post-hoc* tests).

Although there were no differences due to gestational stress for plasma levels of T, DHT, or E_2_ (Table 1), there were differences in plasma levels of 3α-diol [*F*_(1, 44)_ = 3.69, *p* < 0.06]. Gestational stress tended to reduce plasma 3α-diol levels compared to that observed in non-stressed controls (Figure 2). Plasma levels of T, DHT, and 3α-diol were in the ranges reported in the literature of gonadally intact adult male rats (T: 12.0–5.0 ng/ml, DHT: 6.0–3.5 ng/ml; 3α-diol: 15.0–1.5 ng/ml; Frye and Edinger, 2004; Edinger and Frye, 2007a; Frye et al., 2010), but plasma E_2_ levels tended to be higher than a previous study (0.8 pg/ml ± 0.5 s.e.m.; Frye et al., 2010).

**Table 1:** **Plasma and hippocampal levels of testosterone (T) and its aromatized metabolite, estradiol (E_2_)**.

**Endocrine measures**	**Condition**			
	**Control**		**Gestationally-stressed**	
	**No acute stress**	**Acute stress**	**No acute stress**	**Acute stress**
Plasma T (ng/ml)	9.2 ± 3.4	4.2 ± 1.0	7.0 ± 1.7	5.6 ± 1.1
Hippocampus T (ng/mg)	7.1 ± 1.9	5.2 ± 1.4	4.4 ± 0.8	4.6 ± 1.2
Plasma E_2_ (pg/ml)	4.8 ± 1.0	4.0 ± 0.8	4.7 ± 1.1	6.3 ± 1.4
Hippocampus E_2_ (pg/mg)	1.3 ± 0.2	2.6 ± 1.0	1.9 ± 0.8	1.2 ± 0.2

Data are expressed as mean + S.E.M. There were no significant effects of gestational or acute stress, or interactions of these variables, to report for these measures.

There were significant effects of gestational stress for hippocampal levels of DHT [*F*_(1, 44)_ = 5.18, *p* < 0.03], but not T, E_2_, or 3α-diol. Gestational restraint stress significantly reduced hippocampal DHT levels, compared to non-stressed controls (Figure 2). Hippocampus levels of T, E_2_, DHT, and 3α-diol were similar to ranges of levels reported in the literature of gonadally intact adult male rats (T: 4.0–7.0 ng/mg, E_2_: 1.3 pg/mg ± 0.3 s.e.m., DHT: 3.0–1.5 ng/mg; 3α-diol: 5.0–2.7 ng/mg; Frye and Edinger, 2004; Edinger and Frye, 2007a; Frye et al., 2010).

**Figure 3:**
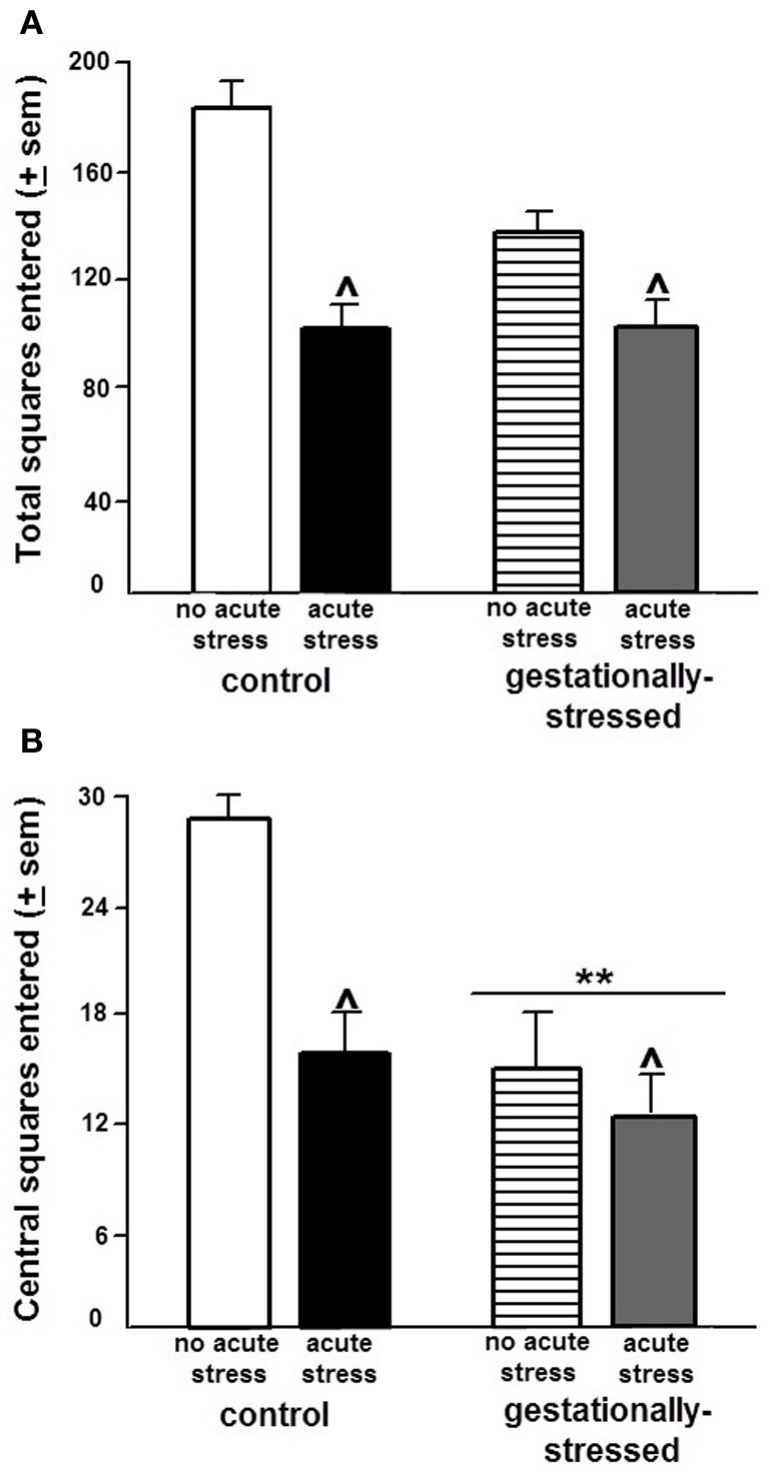
**Open Field Behavior.** Figure depicts total (panel **A**) and central (panel **B**) entries made (mean ± s.e.m.) made in the open field of adult male rats that were gestationally stressed or not, and then restraint stressed, or not, immediately before testing in the open field. ^**^ Above line indicates a significant difference of gestational stress compared to non-gestationally stressed (control) rats (*p* < 0.05 for main effect and Fisher's PLSD *post-hoc* tests). ^∧^ Indicates a significant difference of acute restraint stress compared to no acute stress group (*p* < 0.05 for main effect and Fisher's PLSD *post-hoc* tests). There were no significant interactions between stress variables for performance in the open field.

**Figure 4:**
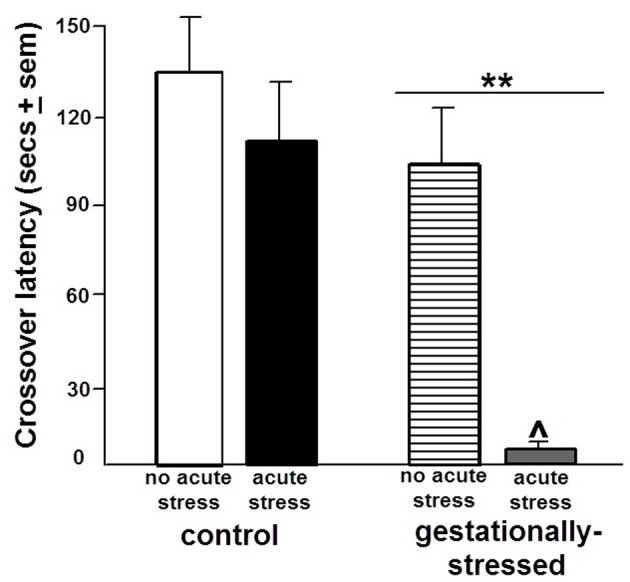
**Inhibitory Avoidance Performance.** Figure depicts the crossover latencies (mean in secs ± s.e.m.) during testing of adult male rats that were gestationally stressed or not, and then restraint stressed, or not, immediately before training in the inhibitory avoidance task. ^**^ Above line indicates a significant difference of gestational stress compared to non-gestationally stressed (control) rats (*p* < 0.05 for main effect and Fisher's PLSD *post-hoc* tests). ^∧^ Indicates a significant difference of acute restraint stress compared to no acute stress group (*p* < 0.05 for main effect and Fisher's PLSD *post-hoc* tests). There was no significant interaction between stress variables for performance in the inhibitory avoidance task.

**Figure 5:**
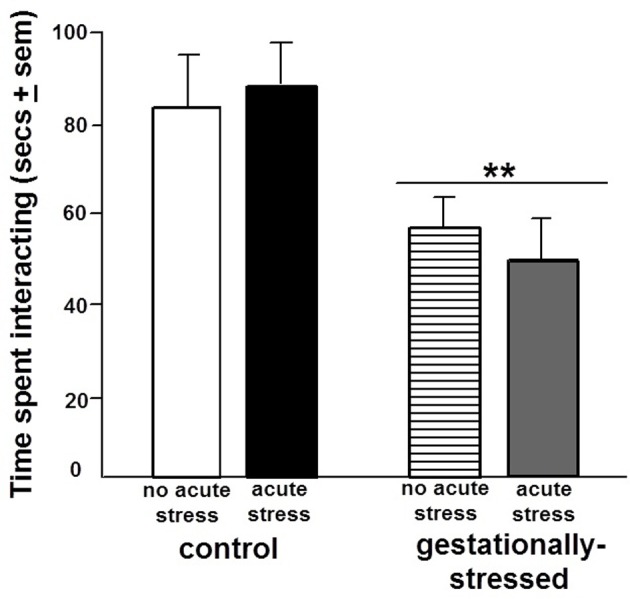
**Social Interaction.** Figure depicts the duration of time spent in social interaction with a conspecific (mean in secs ± s.e.m.) of adult male rats that were gestationally stressed or not, and then restraint stressed, or not, immediately before testing. ^**^ Above line indicates a significant difference of gestational stress compared to non-gestationally stressed (control) rats (*p* < 0.05 for main effect and Fisher's PLSD *post-hoc* tests). There was no significant interaction between stress variables for social interaction.

There were significant effects of gestational stress for behavioral responses in the open field, inhibitory avoidance, and social interaction tasks. There were significant effects of gestational stress for central [*F*_(1, 44)_ = 4.84, *p* < 0.03], but not total, entries in the open field. Gestational stress decreased central open field entries compared to that observed in the non-stress condition (Figure 3). A similar pattern was observed in the inhibitory avoidance task. Rats that were gestationally stressed [*F*_(1, 35)_ = 6.29, *p* < 0.02] had lower crossover latencies in the inhibitory avoidance task compared to non-stressed control rats (Figure 4). Similarly, in the social interaction task, gestationally stressed rats, compared to control rats, spent significantly less time engaging in social interaction with a conspecific [*F*_(1, 44)_ = 7.29, *p* < 0.01; Figure 5].

### Effects of acute restraint stress

The effects of the acute restraint stress paradigm utilized were validated by a demonstrated increase in plasma corticosterone [*F*_(1, 44)_ = 10.43, *p* < 0.01]. Compared to the non-stressed control rats, acute restraint stress significantly increased plasma corticosterone levels (Figure 1).

There were no differences due to acute restraint stress for plasma or hippocampus levels of T, E_2_, or DHT, or plasma 3α-diol levels (Table 1, Figure 2), there were differences in hippocampal levels of 3α-diol [*F*_(1, 44)_ = 10.43, *p* < 0.01]. Rats that were exposed to acute restraint stress had significantly reduced levels of 3α-diol in the hippocampus compared to the non-stressed rats (Figure 2).

There were significant effects of acute restraint stress for behavioral responses in the open field and inhibitory avoidance task. Acute restraint stress decreased total [*F*_(1, 44)_ = 15.36, *p* < 0.01] and central [*F*_(1, 44)_ = 3.80, *p* < 0.05] open field entries compared to the non-stress control condition (Figure 3). Similarly, in the inhibitory avoidance task, acute restraint stress [*F*_(1, 35)_ = 4.25, *p* < 0.04] decreased crossover latencies in the inhibitory avoidance task compared to the non-stress condition (Figure 4). There were no significant effects of gestational stress for behavioral responses in the social interaction (Figure 5) elevated plus maze, paw lick, tailflick, or defensive freezing tasks (Table 2).

**Table 2:** **Behavioral data in the elevated plus maze, pawlick, tailflick, and defensive burying task**.

**Behavioral measures**	**Condition**			
	**Control**		**Gestationally-stressed**	
	**No acute stress**	**Acute stress**	**No acute stress**	**Acute stress**
Elevated plus maze—*open arm time (s)*	25.4 ± 6.0	13.2 ± 4.5	10.3 ± 3.9	13.8 ± 6.6
Pawlick—*front paw latency (s)*	137.3 ± 14.3	125.7 ± 17.0	122.2 ± 10.9	106.2 ± 16.6
Pawlick—*back paw latency (s)*	167.0 ± 7.7	149.0 ± 12.6	149.0 ± 10.3	135.4 ± 15.9
Tailflick—*average latency (s)*	5.9 ± 0.4	5.3 ± 0.3	5.4 ± 0.6	6.1 ± 0.5
Defensive burying—*time spent burying (s)*	101.3 ± 40.4	87.5 ± 41.9	49.0 ± 28.0	46.2 ± 31.1

Data are expressed as mean + S.E.M. There were no significant effects of gestational or acute stress, or interactions of these variables, to report for these measures.

### Interactions between gestational and acute stress

There was a tendency for an interaction between gestational and acute stress to alter plasma DHT levels. Plasma levels of DHT tended to be reduced most greatly among gestationally stressed rats following acute restraint stress [*F*_(1, 44)_ = 3.17, *p* < 0.08], compared to controls. Similarly, there was a significant interaction for gestational and acute restraint stress for plasma 3α-diol levels [*F*_(1, 44)_ = 6.98, *p* < 0.01]. Plasma 3α-diol levels were reduced particularly in rats that were gestationally stressed, and not exposed to restraint stress as adults, compared to non-stressed rats.

Albeit not statistically significant, there was evidence for an interaction between gestational and acute restraint stress for activity of 5α-reductase and 3α-HSD in the hippocampus. Gestationally stressed rats that were exposed to acute stress as adults had the lowest 5α-reductase (2.5 ± 1.2), and highest 3α-HSD (2.7 ± 0.8), conversion ratios, compared to all other groups: gestationally stressed and no acute stress (5α-reductase 3.1 ± 1.0; 3α-HSD 2.2 ± 0.7), no gestational stress and acute restraint stress (5α-reductase 2.9 ± 0.8; 3α-HSD 1.2 ± 0.5), and no restraint stress during gestation or adulthood (5α-reductase 2.9 ± 0.8; 3α-HSD 1.7 ± 0.6).

## Discussion

Our hypotheses that rats exposed to gestational and acute stress may increase corticosterone secretion, alter androgen levels, and produce behavioral inhibition, and gestational stress may potentiate the effects of acute stress exposure in adulthood, was supported in the following ways. Gestational stress increased corticosterone levels, decreased plasma 3α-diol levels, decreased hippocampal DHT levels and produced behavioral inhibition in the open field, inhibitory avoidance, and social interaction tasks. Acute restraint stress increased corticosterone levels, decreased hippocampal 3α-diol levels and produced behavioral inhibition in the open field and inhibitory avoidance task. There was evidence that gestational stress exposure altered later neuroendocrine, but not behavioral, responses of acutely restraint stressed rats. Plasma levels of DHT and 3α-diol were lowest, hippocampal 5α-reductase activity was lowest, and hippocampal 3α-HSD activity was highest among gestationally stressed rats that were acutely restraint stressed. No group differences were noted for plasma or hippocampal levels of T, or its aromatized metabolite. Together these data show that gestational and acute restraint stressors have actions to increase HPA responding as measured by plasma corticosterone, alter 5α-reduced T metabolite levels in plasma and hippocampus, and produce behavioral inhibition. Further, gestational stress may impose organizational effects to alter androstane neurosteroid responses to acute stress exposure in adulthood.

The present study confirms and extends the previously reported effects of gestational stress to produce behavioral inhibition and alter functional effects of androgens in the open field and inhibitory avoidance task. In the present study, gestational stress reduced central entries made in the open field. In previous studies, gestationally stressed male rodents have greater depression-like behavior in the forced swim or sucrose anhedonia test (Frye and Wawrzycki, 2003; Mueller and Bale, 2008). As well, rats that were gestationally stressed had poorer performance than did their non-stressed controls in the inhibitory avoidance task. This pattern confirms previous reports on the role of HPA dysregulation for cognitive and/or emotional memory task performance of rats. In support, gestationally stressed female rats have poorer performance in the inhibitory avoidance task compared to their non-stressed controls (Walf and Frye, 2007) and gestationally stressed male rats have poorer spatial performance (Lemaire et al., 2000; Zagron and Weinstock, 2006). Gestational stress reduced time spent in social interaction with a conspecific. Other studies have demonstrated gestational challenges alter social interaction and reproductive behaviors (Ward et al., 1994, 1996, 1999; Lee et al., 2007). In the present study, there were no effects of acute restraint stress for social interaction. These data suggest that there may be differences in the behavioral outcomes of restraint during early development versus later in life for this androgen-sensitive behavior. A question is whether more robust differences for social behavior would have been observed in more challenging and androgen-sensitive situations, such as mating and/or agonistic encounters (DeBold and Miczek, 1981; Lumia et al., 1994; McGinnis, 2004). Individual differences in mating responses and subsequent central production of androstane neurosteroids in the brain mediate anxiety-responding of adult male rats (Edinger and Frye, 2007a). In the present study, there was reduced androstane neurosteroids and social interaction among rats that were gestationally stressed. A question to address in future studies is the extent to which gestational stress may have pervasive effects to alter mating and mating-induced neurosteroidogenesis among males.

The present study confirms and extends the previously reported pattern of acute stress altering steroid-mediated responding among rats. In the present study, we found that male rats that were restraint stressed for 20 min before behavioral assessments had behavioral inhibition, as evidenced by fewer total and central square entries made, compared to their non-stressed counterparts. We have observed a similar pattern of behavioral inhibition in the open field and elevated plus maze among adult, ovariectomized, E_2_-primed following 20 min of restraint stress, compared to their non-stressed counterparts (Walf and Frye, 2005). Interestingly, in the present study, the effects of acute stress were more robust in the open field than in other anxiety tasks assessed, the elevated plus maze or defensive burying task. Although differences between these anxiety tasks were not anticipated, the timing of when rats were tested in these tasks suggests that the greatest amount of behavioral inhibition occurred immediately following acute restraint stress. Rats were exposed to acute restraint stress immediately before testing in the open field; whereas, stressor exposure was approximately 5 and 20 min before testing in the elevated plus maze and defensive freezing task, respectively. Additionally, rats that were acutely restraint stressed before training in the inhibitory avoidance task demonstrated memory impairments in this task when tested 24 h later. Although we did not measure corticosterone following training, we predict that corticosterone levels were high among restraint stressed rats during training and in the period afterward, thus, interfering with memory consolidation. Additionally, restraint stress reduced levels of 3α-diol in the hippocampus. Previous studies have demonstrated that 3α-diol has actions in the hippocampus to improve cognitive function and decrease anxiety-like responding of male rats (Edinger and Frye, 2004, 2007b; Frye and Edinger, 2004; Frye et al., 2008, 2010). Thus, restraint stress produced behavioral inhibition in the open field task and performance deficits in the inhibitory avoidance task, and reduced hippocampus levels of 3α-diol.

The present data show that gestational stress can have pervasive effects on adult responding to an acute restraint stressor. These effects were apparent for rats' neuroendocrine responses, rather than behavioral effects. Rather than alterations in T or E_2_ levels among male rats in the present study, salient reductions in plasma levels of DHT and 3α-diol were observed for gestationally stressed rats exposed to acute restraint stress. As well, these results of lower levels of DHT and 3α-diol suggest that stress exposure during gestation and adulthood may have reduced expression or activity of the requisite enzymes, 5α-reductase and 3α-HSD, respectively. Although expression and activity of 5α-reductase and 3α-HSD were not measured directly, calculated conversion ratios suggested a pattern of decreased 5α-reductase and increased 3α-HSD activity among gestationally stressed rats that were acutely restraint stressed as adults. Enzymes, such as the 5α-reductase isozymes, are involved in organizational effects of steroids on the brain during early development, and there are sex differences in adulthood as to how androgens modify these enzymes (Torres and Ortega, 2003, 2006). Neonatal manipulations of T irreversibly program the expression of these enzymes that convert T to DHT and 3α-diol in the liver of rats (Gustafsson and Stenberg, 1974a,b). The role of stressors for regulating 5α-reductase and other steroidogenic enzymes, and their neurosteroid products, throughout development has been described. Prenatal immobilization stress on gestational days 15–18 is associated with initial decreases in 5α-reductase activity in the cerebral cortex and hypothalamus of PND 1 male pups, but elevated 5α-reductase activity in the cortex, hippocampus, and hypothalamus on PND 5 (Ordyan and Pivina, 2005). Another model of early life stress, isolation rearing, for 5–8 weeks reduces expression of 5α-reductase and levels of 3α,5α-THP in the nucleus accumbens and medial prefontal cortex of male rats (Bortolato et al., 2011). Among PND 7 male and female rats, high expression of 3 alpha-HSD mRNA was found, which is coincident with the stress hyporesponsive period in the rat (Mitev et al., 2003). Conversely, acute swim or environmental stress among adult male rats increases prefrontal cortex expression of 5α-reductase (Sánchez et al., 2008, 2009). In our laboratory, social challenges, such as paced mating, reliably increase production of the pregnane neurosteroid, 3α,5α-THP, in the midbrain, hippocampus, and prefrontal cortex of female rats (Frye et al., 2007). As such, the present results may be related to the pervasive effects of acute and chronic stressors on activity and/or expression of metabolism enzymes. Moreover, 3α-diol is a positive allosteric modulator of GABA/benzodiazepine receptor complexes (Gee, 1988), and like the pregnane neurosteroid, 3α,5α-THP, may be released with stressors to dampen the HPA response and restore homeostasis (Erskine and Kornberg, 1992; Patchev et al., 1994, 1996; Frye, 2009). There is recent evidence for 3α-diol to reduce HPA hyper-responsiveness to a physical, stressor, IL-1β administration, of gestationally stressed male rats (Brunton and Russell, 2010). Together, these data further provide evidence supporting a role of neurosteroids as modulators of the HPA (Purdy et al., 1991; Patchev et al., 1994, 1996; Guo et al., 1995). Future studies will further investigate this notion that some of these behavioral deficits with stress could be related to differences in capacity for androgens to be metabolized.

In summary, the present study demonstrated that gestational and acute restraint stress increased corticosterone secretion, reduced levels of androstane neurosteroids, and produced behavioral inhibition of adult male rats. It is important to determine how sex/gender and gonadal hormones may mitigate stress responses following early life adversity because these factors influence the individual's developmental trajectory and pathophysiological states. Neuropsychiatric disorders, such as anxiety, depression, and PTSD, are stress-related disorders that are influenced by sex/gender and gonadal hormones. Indeed, neurodegeneration, as can occur with aging or disease, can be exacerbated by stress and influenced by sex/gender and gonadal hormones. Of clinical significance is that some males may particularly be sensitive to stressors in adulthood when androgen levels are perturbed. Examples of this may be “roids rage” and/or post-finasteride syndrome. The understanding of these pathophysiological states is important to reveal the etiology of disorders, but also for elucidating the possible mechanisms of the normative state, which may be influenced by interactions between adrenal and gonadal hormones, and their metabolism, during different developmental periods.

### Conflict of interest statement

The authors declare that the research was conducted in the absence of any commercial or financial relationships that could be construed as a potential conflict of interest.
